# CGG-repeat dynamics and *FMR1* gene silencing in fragile X syndrome stem cells and stem cell-derived neurons

**DOI:** 10.1186/s13229-016-0105-9

**Published:** 2016-10-06

**Authors:** Yifan Zhou, Daman Kumari, Nicholas Sciascia, Karen Usdin

**Affiliations:** 1 Section on Gene Structure and Disease, Laboratory of Molecular and Cellular Biology, National Institute of Diabetes, Digestive and Kidney Diseases, National Institutes of Health, Bethesda, MD USA; 2 Present Address: Laboratory of Genome Integrity, National Cancer Institute, Bethesda, MD USA

**Keywords:** Fragile X syndrome, Repeat expansion mutation, Repeat-mediated gene silencing, Repeat contractions, Stem cells

## Abstract

**Background:**

Fragile X syndrome (FXS), a common cause of intellectual disability and autism, results from the expansion of a CGG-repeat tract in the 5′ untranslated region of the *FMR1* gene to >200 repeats. Such expanded alleles, known as full mutation (FM) alleles, are epigenetically silenced in differentiated cells thus resulting in the loss of FMRP, a protein important for learning and memory. The timing of repeat expansion and *FMR1* gene silencing is controversial.

**Methods:**

We monitored the repeat size and methylation status of *FMR1* alleles with expanded CGG repeats in patient-derived induced pluripotent stem cells (iPSCs) and embryonic stem cells (ESCs) that were grown for extended period of time either as stem cells or differentiated into neurons. We used a PCR assay optimized for the amplification of large CGG repeats for sizing, and a quantitative methylation-specific PCR for the analysis of *FMR1* promoter methylation. The *FMR1* mRNA levels were analyzed by qRT-PCR. FMRP levels were determined by western blotting and immunofluorescence. Chromatin immunoprecipitation was used to study the association of repressive histone marks with the *FMR1* gene in FXS ESCs.

**Results:**

We show here that while *FMR1* gene silencing can be seen in FXS embryonic stem cells (ESCs), some silenced alleles contract and when the repeat number drops below ~400, DNA methylation erodes, even when the repeat number remains >200. The resultant active alleles do not show the large step-wise expansions seen in stem cells from other repeat expansion diseases. Furthermore, there may be selection against large active alleles and these alleles do not expand further or become silenced on neuronal differentiation.

**Conclusions:**

Our data support the hypotheses that (i) large expansions occur prezygotically or in the very early embryo, (ii) large unmethylated alleles may be deleterious in stem cells, (iii) methylation can occur on alleles with >400 repeats very early in embryogenesis, and (iv) expansion and contraction may occur by different mechanisms. Our data also suggest that the threshold for stable methylation of FM alleles may be higher than previously thought. A higher threshold might explain why some carriers of FM alleles escape methylation. It may also provide a simple explanation for why silencing has not been observed in mouse models with >200 repeats.

**Electronic supplementary material:**

The online version of this article (doi:10.1186/s13229-016-0105-9) contains supplementary material, which is available to authorized users.

## Background

Fragile X syndrome (FXS) is the most common heritable form of intellectual disability and a common cause of autism spectrum disorder [1]. FXS results from the absence of functional FMRP, the *FMR1* gene product. Most cases of FXS result from the epigenetic silencing of the *FMR1* gene that occurs when a CGG-repeat tract in the 5′ untranslated region (UTR) expands to >200 repeats. Such alleles, known as full mutation (FM) alleles, arise by expansion of premutation (PM) alleles with 55–200 repeats when these alleles are maternally transmitted. Rare individuals are known who carry an unmethylated FM (UFM) [2–10]. Such individuals show few, if any, symptoms of FXS; although, they are at risk for the neurodegenerative disorder, Fragile X-associated tremor/ataxia syndrome (FXTAS), that is seen in PM carriers [9–11]. How UFM alleles escape methylation is unknown.

The timing of expansion is controversial. Male FM carriers do not have FM alleles in their sperm [12] and do not transmit FM alleles to their offspring [13, 14]. One interpretation of these observations is that expansion to the FM is a post-zygotic event that spares the male germ line [12]. However, it is also possible that expansion occurs prior to the differentiation of the germ line with selection against FM alleles in sperm. Since FM alleles are difficult to replicate [15], selection may result from the requirement for rapid cell division during spermatogenesis. The fact that a number of embryonic stem cells (ESCs) have been characterized in which expansion into the FM range is already present [16–18] lends support to the idea that expansion occurs either prezygotically or in the early embryo. Furthermore, the maternal age effect on expansion risk suggests that most expansions may be prezygotic [19]. However, the somatic mosaicism seen in many FXS patients raises the possibility that expansion also occurs in the early embryo. An early embryonic origin for expansions would be consistent with work in other repeat expansion diseases where expansion has been reported in both patient-derived induced pluripotent stem cells (iPSCs) [20–22] and embryonic stem cells (ESCs) [23, 24]. Expansion in these cells has been attributed to elevated levels of the mismatch repair proteins that are known to be required for expansion in different mouse models [25–28].

Methylated alleles are known to be more stable than unmethylated ones [29]. Expansions in a FX PM mouse model are only seen when the PM allele is on the active X chromosome in females [30] and a retrospective examination of data from women who carry the PM [31] suggests that the same is true in humans. Thus, expansions require the expansion-prone allele to be transcribed or present in a region of open chromatin. Whether expansions and contractions arise by the same mechanism is also unclear. The fact that the AGG interruptions sometimes found in the *FMR1* gene reduces expansion risk but does not affect contraction risk suggests that the mechanisms may be different [32].

When *FMR1* gene silencing occurs is also the subject of some debate. One of the first FX ESC lines to be described, HEFX, was transcriptionally active [16]. However, differentiation into teratomas only resulted in ~5 % methylation. Studies of other FX ESCs led to the suggestion that gene silencing only occurs late in the neuronal differentiation process [18]. However, recent work has demonstrated that many FX ESCs already show full or partial silencing of the FM allele [17, 33]. This raises the possibility that silencing occurs in the very early embryo. Whether the silencing seen on neuronal differentiation reflects an additional opportunity for gene inactivation is unclear. Resolving the question of the timing of expansion and gene silencing is relevant both for our understanding of the consequences of the FX mutation for affected individuals and for our understanding of the mechanisms involved.

## Methods

### Cell lines and culture

The iPSC line SC120 (clone 14) was provided by Dr. P. Schwartz, Children’s Hospital of Orange County Research Institute, California, and was derived from a fragile X premutation fibroblast cell line (SC120) using the lentivirus method [34]. The iPSC line HT14 (clone 3) was generated from a fragile X syndrome (FXS) patient fibroblast cell line, C10147 [35], using an integration free protocol [36]. The control hESC line, H1 (WA01, WiCell Research Institute, Madison, WI), was obtained from Dr. Barbara Mallon, NIH Stem Cell Unit (Bethesda, MD) and the FXS hESC line, WCMC37 [18], was obtained from Nikica Zaninovic, Weill Cornell Medical College of Cornell University (New York, NY). All stem cells were grown feeder-free on Matrigel™ (BD Biosciences, Franklin Lakes, NJ) -coated plates in mTeSR™1 medium (STEMCell Technologies, Vancouver, British Columbia, Canada). Cells were passaged using either StemPro® Accutase® (Thermo Fisher Scientific, Waltham, MA) or EDTA [37]. For isolating individual lineages from stem cells, single colonies were picked and expanded in mTeSR™1 medium. Cell viability and proliferation in the hESCs were measured by the AlamarBlue® cell viability assay reagent (Thermo Fisher Scientific) as per manufacturer’s instructions. Briefly, 7000 cells were plated in triplicate in 96-well plates in 100-μl medium and allowed to attach overnight. The medium was replaced with fresh 100-μl medium and 10 μl of Alamarblue® reagent was added. This was noted as time 0. The fluorescence was read at 590 nm in a Synergy™ 2 plate reader (BioTek, Winooski, VT) at 2, 4, 8, 12, and 24 h. The reading at each time was divided by the reading at time 0 to correct for the differences in the starting cell number in each well. The 37D cells were treated with either 1 mM caffeine or 10 μM ATM kinase inhibitor KU55933 for 24 h and then grown in drug-free medium for the next 3 days. Cells were passaged on the third day and treated with the drug again for 24 h for a total of three treatments. After the last treatment, cells were grown for 3 days in drug-free medium and harvested for DNA.

### Neuronal differentiation

The SC120 cells were differentiated into post-mitotic neurons using the previously described dual SMAD inhibition method [38] with some modifications. Briefly, the iPSCs were dissociated into single cells using StemPro®Accutase® and re-plated into ultra low-attachment dishes (Corning, Corning, NY) in KnockOut™ serum replacement (Thermo Fisher Scientific) based medium with 20 ng/ml bFGF (R&D Biosystems, Minneapolis, MN), 20 μM ROCK inhibitor (Stemgent, San Diego, CA), 10 μM SB431542 (Stemgent), and 0.2 μM LDN193189 (Stemgent). On the third day, the embryoid bodies were transitioned to neural induction medium (NIM) [DMEM/F12, 1X GlutaMAX™, 1X non-essential amino acids (NEAA), 1X N2 supplement (all reagents from Thermo Fisher Scientific)] supplemented with 0.2 μM LDN193189 and 10 μM SB431542. On day 6, cells were transitioned to NIM media plus 20 ng/ml bFGF. The neural aggregates were grown in this medium for another 4 days with media change every other day. After 10 days of culture in suspension, the neural aggregates were dissociated and plated on dishes coated with Geltrex® (Thermo Fisher Scientific) and cultured in NIM media supplemented with 20 ng/ml bFGF for another 7 days. Neural rosettes were purified using the STEMdiff™ neural rosette selection reagent (STEMCell Technologies) as per the manufacturer’s instructions. The purified neural progenitor cells (NPCs) were plated on Geltrex-coated dishes and cultured in neural stem cell (NSC) expansion medium (neurobasal medium, 1X B27 supplement, 1X NEAA, 1X GlutaMAX™ (all from Thermo Fisher Scientific) and 20 ng/ml bFGF). For terminal differentiation into neurons, NPCs were plated on polyornithine (Sigma-Aldrich, St. Louis, MO) and laminin (Roche Applied Science, Mannheim, Germany) -coated dishes in NSC expansion medium with 1X N2 supplement without bFGF. The 37D cells were differentiated into neurons as described previously [18]. Briefly, ESCs were cultured in mTeSR™1 on Matrigel™ to 90 % confluency, followed by culture in initial differentiation medium [mTeSR™1 plus 10 μM SB431542 and 250 ng/mL Noggin (R&D Biosystems)] for 5 days. Beginning day 6, increasing N2 (25, 50, 75 %) was added to the neural differentiation medium every other day. On day 12, neural rosettes were disaggregated using StemPro®Accutase® and plated onto polyornithine and laminin-coated plates in neural differentiation medium [DMEM/F-12 with GlutaMAX™, 1X N2, 1X B27, 20 ng/mL BDNF (R&D Biosystems), 20 ng/mL GDNF (Thermo Fisher Scientific), 1 mM cAMP (Sigma-Aldrich), 200 nM ascorbic acid (Sigma-Aldrich)]. Half of the neural differentiation medium was changed every other day.

### Immunostaining

Cells were grown in 24-well plates and fixed in 4 % PFA. Prior to immunostaining, cells were permeabilized and blocked with 3 % Triton X-100 (Sigma-Aldrich), 10 % normal goat serum (Thermo Fisher Scientific) in PBS for 1 h at room temperature, followed by incubation with primary antibodies diluted in 1 % BSA (Sigma-Aldrich), 1 % normal goat serum, and 0.3 % Triton X-100 in PBS overnight at 4 °C. The following antibodies were used: Oct4 (Stemgent, cat # 09-0023, 1:1000), Nanog (EMD Millipore, cat # MABD24, 1:500), Nestin (EMD Millipore, cat # AB5922, 1:1000), MAP2 (Sigma-Aldrich, 1:500, cat # M2320), and β-III tubulin/TuJ1 (Covance Inc., Chantilly, VA, 1:2000). For FMRP immunostaining, the cells were blocked and permeabilized with 5 % normal goat serum and 0.1 % saponin (Sigma-Aldrich) for 1 h at room temperature followed by incubation with 1:500 dilution of FMRP antibody (Biolegend, cat # MMS-5231) in 1 % BSA and 0.1 % saponin in PBS overnight at 4 °C. For Sox1 immunostaining, cells were permeabilized with 0.2 % Triton X-100 in PBS for 15 min at room temperature, neutralized with 100 mM glycine for 5–10 min, followed by blocking in 3 % BSA for 45 min. The cells were then incubated with Sox1 antibody (R&D Biosystems, cat # A11055) diluted 1:50 in 3 % BSA, 0.1 % Tween 20 (Sigma-Aldrich) in PBS overnight at 4 °C. The cells were then incubated with appropriate secondary antibodies labeled with either alexa-488 or alexa-555 (Thermo Fisher Scientific) for 1 h at room temperature. The nuclei were counterstained with DAPI for 15 min at room temperature and the images acquired using EVOS®FL Microscope (Thermo Fisher Scientific).

### DNA methods

DNA was isolated from pluripotent stem cells and neurons at indicated times by the salting out method [39], and the CGG-repeat number in SC120 iPSCs and WCMC37 hESCs and lineages derived from these cells were analyzed by RPT-PCR and MS_RPT-PCR respectively as described before [40]. Briefly, 300 ng of genomic DNA was digested with either Fast-HindIII (Thermo Fisher Scientific) or HindIII with HpaII (33 units/μg DNA) (New England Biolabs, Ipswich, MA) overnight in 22.5 μl volume containing 50 mM Tris-HCl (pH 8.9), 1.5 mM MgCl_2_, 22 mM (NH_4_)_2_SO_4_, and 0.2 % Triton X-100. Total 5 μl of digested DNA from both samples were processed for the repeat PCR with 15-μl PCR reaction mix containing a final composition of 50 mM Tris-HCl (pH 8.9), 1.5 mM MgCl_2_, 22 mM (NH_4_)_2_SO_4_, 0.2 % Triton X-100, 0.5 μM of the primers Not_FraxC (5′-AGTTCAGCGGCCGCGCTCAGCTCCGTTTCGGTTTCACTTCCGGT-3′) and Not_FraxR4 (5′-CAAGTCGCGGCCGCCTTGTAGAAAGCGCCATTGGAGCCCCGCA-3′), 2.5 M betaine (Sigma-Aldrich), 2 % DMSO (Sigma-Aldrich) and 0.27 mM each dNTP (New England Biolabs), and 0.4 units of Phusion® DNA polymerase (New England Biolabs). The samples were then loaded onto a preheated (>70 °C) PCR block (C1000-Touch, Bio-Rad, Hercules, CA) and subjected to the following cycles of heating and cooling: 98 °C for 3 min, 30× (98 °C for 30 s, 64 °C for 30 s, 72 °C for 210 s) and 72 °C for 10 min. The PCR products were resolved on TAE-1 % agarose gels and visualized with ethidium bromide according to standard procedures. For samples analyzed on capillary electrophoresis, Not FraxR4 primer was FAM labeled. For the HT14 iPSCs, the CGG-repeat size was determined as previously described [41] except that 4, 7, 2′, 4′, 5′, 7′-hexachloro-6-carboxyfluorescein (HEX)-labeled primer frax-F (5′-AGCCCCGCACTTCCACCACCAGTCTCCTCCA-3′) and primer frax-C (5′-GCTCAGCTCCGTTTCGGTTTCACTTCCGGT-3′) were used. This primer pair produces a PCR product that contains the repeat and 121 bp of DNA from the upstream flank and 100 bp from the downstream flank for a total of 221 bp of the flanking DNA. The *FMR1* promoter methylation was assessed by quantitative methylation-specific PCR (qMS-PCR) essentially as described before [40]. Briefly, 300 ng of DNA was sonicated into <500-bp fragments using a Bioruptor® (Diagenode, Denville, NJ) and digested overnight with HpaII or mock digested. Quantitative PCR using 2 μl of the mock-digested or HpaII-digested DNA was then carried out in 5 replicates using Power SYBR® green master mix on StepOnePlus™ Real-Time PCR machine (Thermo Fisher Scientific) in a final volume of 20 μl. The percentage of DNA methylation was determined by averaging the results of five technical replicates and calculating the difference in the Ct values of the digested and mock-digested samples using the ∆∆Ct method. *FMR1* promoter region was amplified using primers *FMR1* ex 1 (F) (5′-GAACAGCGTTGATCACGTGAC-3′) and *FMR1* ex 1 (R) (5′-GTGAAACCGAAACGGAGCTGA-3′). The efficiency of HpaII digestion was assessed by amplification of an unmethylated region of *GAPDH* using primers *GAPDH* exon1 (F) (5′-TCGACAGTCAGCCGCATCT-3′) and *GAPDH* intron1 (R) (5′-CTAGCCTCCCGGGTTTCTCT-3′). Additionally, DNA methylation at 22 CpG residues in the *FMR1* promoter in WCMC37 p45 cells was also analyzed by pyrosequencing of bisulfite-converted DNA by EpigenDx, Inc. (Hopkinton, MA) using assays ADS1451-FS1 and ADS1451-FS2.

### RNA methods

Total RNA was isolated using Trizol™ (Thermo Fisher Scientific) as per the manufacturer’s instructions. Three hundred nanograms of total RNA was used for cDNA synthesis using VILO™ master mix (Thermo Fisher Scientific) in 20-μl reaction volume. *FMR1* and *β-glucuronidase (GUS)* mRNAs were quantified by real-time PCR using TaqMan® Fast Universal PCR master mix (Thermo Fisher Scientific) and TaqMan probe-primer pair (FAM™ for *FMR1* and VIC® for *GUS*, Thermo Fisher Scientific) on StepOnePlus™ Real-Time PCR machine (Thermo Fisher Scientific).

### Western blotting

For making total cell lysates, cells were harvested in the growth medium and pelleted at 250 × g for 6 min. The cell pellet was washed with ice-cold PBS supplemented with 1X protease inhibitor cocktail (Sigma-Aldrich) and 1X phosphatase inhibitor (Sigma-Aldrich). The pellet was then resuspended in the lysis buffer (10 mM Tris Cl pH 7.5, 1 mM EDTA pH 8.0, 1 % Triton X-100, 1X protease inhibitor cocktail, and 1X phosphatase inhibitor) and incubated on ice for 10 min followed by sonication to solubilize proteins and shear the DNA. The amount of protein in the lysate was quantified using Bio-Rad Protein Assay Dye Reagent Concentrate (Bio-Rad Laboratories, Inc., Hercules, CA) as per manufacturer’s protocol. Proteins were heated for 10 min at 70 °C in LDS sample buffer (Thermo Fisher Scientific) before running on the gel. Ten micrograms of lysates were run on NuPAGE™ 3–8 % Tris-Acetate gels (Thermo Fisher Scientific) and transferred to nitrocellulose membrane using the iBlot transfer apparatus (Thermo Fisher Scientific) as per the manufacturer’s instructions. Membranes were blocked in 5 % membrane blocking agent (GE Healthcare Bio-Sciences, Pittsburgh, PA) diluted in TBST for 1 h at room temperature. The blot was then incubated with specific primary antibodies, diluted in blocking solution, overnight at 4 °C. Primary antibodies and their dilutions were as follows: FMRP (EMD Millipore, cat # MAB 2160) 1:2000, MSH2 (Abcam, Cambridge, MA, cat # Ab 70270) 1:10,000, MSH3 (Santa Cruz Biotechnology, Inc., Dallas, TX, cat # sc-271079) 1:1000, MSH6 (BD Biosciences, cat # BD610918) 1:1000, β-actin (Abcam, cat # ab8226) 1:5000. HRP-labeled secondary antibodies were used at a dilution of 1:5000. The detection was done using the ECL™ Prime western blotting detection reagent (GE Healthcare Bio-Sciences) and imaged using Fluorchem™ M imaging system (Proteinsimple, Santa Clara, CA). The blot was then probed with antibody against β-actin, which was used as a loading control.

### Chromatin immunoprecipitation (ChIP) assay

To prepare chromatin for immunoprecipitation, cells were fixed with 1 % formaldehyde for 10 min at room temperature and lysed as per the kit manufacturer’s instructions. The chromatin was sonicated into <500-bp fragments using a Bioruptor® (Diagenode, Denville, NJ). ChIP assays were performed using a ChIP assay kit from EMD Millipore as described previously [42]. Real-time PCRs on the immunoprecipitated DNAs were carried out in triplicate in 20-μl final volume using the Power SYBR™ Green PCR master mix (Thermo Fisher Scientific) and 200 nM of each primer and 2 μl of DNA. For amplification of *FMR1* exon1, the primer pair Exon1-F (5′-CGCTAGCAGGGCTGAAGAGAA-3′) and Exon1-R (5′-GTACCTTGTAGAAAGCGCCATTGGAG-3′) was used. This primer pair amplifies the region +236 to +312 relative to the transcription start site. *GAPDH* was amplified with primers *GAPDH* exon1 (F) and *GAPDH* intron1 (R). For quantitation, the comparative threshold (Ct) method was used. Enrichment over 5 % of input was calculated and was normalized to *GAPDH*.

## Results and discussion

### Contractions but no expansions are seen in stem cells and in stem cell-derived neurons

**Fig. 1:**
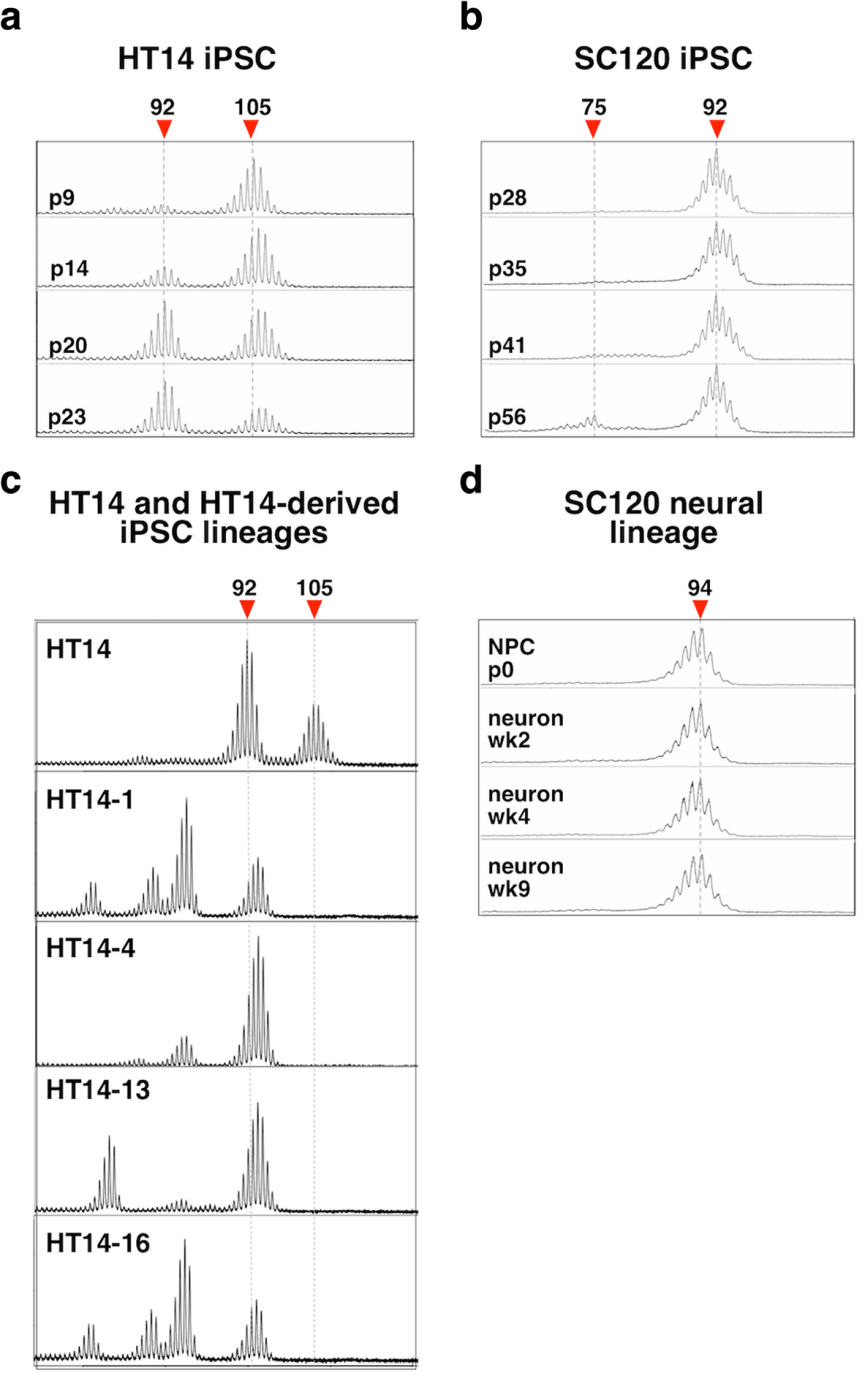
CGG-repeat instability in iPSCs from PM carriers. CGG-repeat size analysis was done by RPT-PCR followed by capillary electrophoresis as described in the “Methods” section for the hiPSCs HT14 (**a**) and SC120 iPSCs (**b**), individual lines derived from HT-14 iPSCs (**c**) and neuronal cells derived from SC120 iPSC (**d**). See Additional file 1: Figure S1a for pluripotency marker staining for these cells and Additional file 1: Figure S1b for neuronal differentiation of SC120 cells

We have previously shown that relatively limited CGG expansion is seen in human lymphoblastoid cells or brains of premutation carriers [41]. Since extensive expansions are seen in iPSCs from patients with other repeat expansion diseases [43], we examined CGG instability in two male PM iPSC lines, HT14 and SC120. Since FXS affects males, should expansion occur in the early embryo, expansion in male stem cells might be observed. HT14 had ~105 CGG repeats at passage 9 (p9), with a small fraction of cells having ~92 repeats, while SC120 had ~92 CGG repeats at p28 (Fig. 1). Both lines were positive for key pluripotency markers (Additional file 1: Figure S1a). Notably, despite the lack of DNA methylation (Additional file 1: Figure S1c), the level of FMRP expression was much lower in SC120 iPSCs than in H1 hESCs with a normal *FMR1* allele (Additional file 1: Figure S1d–e). Thus, even 92 repeats significantly reduces *FMR1* translation in stem cells, a factor that may contribute to pathology seen in PM carriers.

Both iPSC lines were grown for extended times in culture and the CGG-repeat length monitored by PCR and capillary electrophoresis as described in the “Methods” section. No expansions were seen (Fig. 1) despite the fact that such alleles are associated with ~100 % risk of expansion into the full mutation range on maternal transmission [32]. In the HT14 cell line, the smaller allele became more prominent with time while a contraction became apparent in the SC120 cell line (Fig. 1). An increase of 2 repeats was seen during the transition from SC120 iPSCs to neuronal progenitor cell (NPC) stage that may reflect heterogeneity in the original cell line and the repeat remained unchanged on further differentiation into neurons up to 9 weeks in culture. Plating of HT14 cells at low density resulted in lineages with increased representation of contracted alleles (Fig. 1c) Some of these lineages shared a similar set of contracted alleles (e.g., HT14-1 and HT14-16) suggesting that they may have been present at low levels in the parent culture and that the stress associated with growth at low density may have favored the growth of cells with smaller repeat numbers. This stress may be related to the fact that the repeats impede DNA replication [15], an effect that may be particularly problematic when rapid cell division is required.

**Fig. 2:**
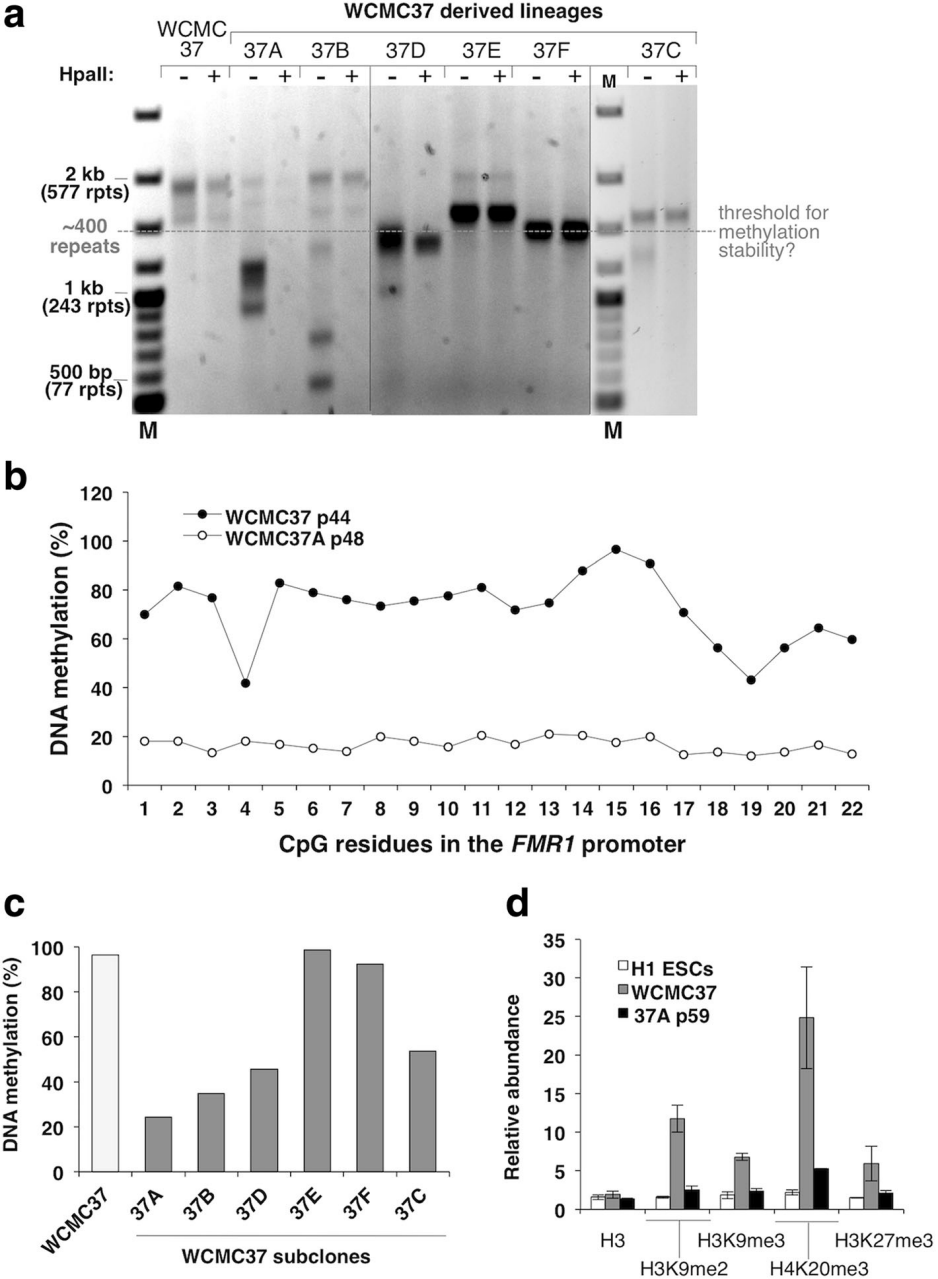
CGG-repeat instability in FX ESCs. **a** CGG-repeat size and methylation analysis for the WCMC37 ESCs and the individual lineages derived from it was done by MS_RPT-PCR followed by agarose gel electrophoresis as described in the “Methods” section. The “+” and “−“ signs indicate the presence or absence of predigestion by the methylation-sensitive restriction enzyme, HpaII. M, 100-bp DNA size ladder and rpts, CGG repeats. **b** Pyrosequencing analysis of DNA methylation in the *FMR1* promoter of WCMC37 and 37A cells at passage 44 and 48, respectively. **c** qMS-PCR analysis of DNA methylation in the *FMR1* promoter of the samples analyzed in **a**. The extent of methylation was determined by the ∆∆Ct method and the individual technical replicates varied by <0.3 Ct. **d** The abundance of total histone H3, H3K9me2, H3K9me3, H3K27me3, and H4K20me3 in the *FMR1* exon1 region is shown relative to *GAPDH*. Data shown are an average of two independent experiments and *error bars* represent standard deviation. The 37A line at passage 59 (p59) showed the presence of mostly active *FMR1* alleles with only 15 % DNA methylation, consistent with the low levels of repressive histone marks on the *FMR1* exon1 compared to WCMC37 cells that carry a silenced *FMR1* allele. The passage number of the cells used in panels **a** and **c** are as follows: WCMC37 p44, 37A p48, 37B p53, 37C p55, 37D p55, 37 F p55, 37E p55. See Additional file 1: Figure S1a for pluripotency marker staining in WCMC37 cells

The high expansion frequency seen in Friedreich ataxia (FRDA) and myotonic dystrophy type 1 (DM1) iPSCs has been attributed to the elevated levels of the mismatch repair proteins important for expansion in stem cells [20, 43]. However, the level of these proteins in PM iPSCs was comparable to that seen in H1 ESCs (Additional file 1: Figure S1f) that have levels of these proteins similar to those in FRDA hiPSCs [43]. Since the PM iPSCs may have repeat numbers too small to show extensive expansion over the relatively short experimental time frame, we acquired an NIH-approved FX ESC line, WCMC37, that was previously reported to carry an unmethylated, active FM allele with ~450 CGG repeats [18]. Analysis of alleles with this number of repeats is difficult because amplification through the repeats is impaired by the secondary structures formed by the repeat [44]. In addition, unmethylated alleles show a high degree of size heterogeneity [29]. It is thus challenging to get an accurate representation of the distribution of repeat sizes and methylation status in a population of cells. We therefore analyzed these cells using a methylation-sensitive repeat (MS_RPT)-PCR assay we developed that accurately reflects the proportion of a wide range of allele sizes and their methylation status, together with a second more quantitative promoter methylation assay, the qMS-PCR assay, that does not involve amplification through the repeat [40]. The WCMC37 ESCs contained a mixture of alleles with ~410–550 repeats (Fig. 2a). These alleles were resistant to predigestion with the methylation-sensitive enzyme HpaII prior to PCR (Fig. 2a). Pyrosequencing (Fig. 2b) and the qMS-PCR assay (Fig. 2c) showed ~ 100 % methylation of the *FMR1* promoter. WCMC37 cells also showed elevated levels of the repressive histone marks (Fig. 2d) present on silenced alleles in differentiated cells [45] and *FMR1* mRNA levels were ~5 % of normal. Immunostaining with an FMRP antibody showed that very little FMRP is produced in these cells (Additional file 1: Figure S1d). An average of 95 % of cells were positive for Nanog and 97 % were positive for Oct4 (Additional file 1: Figure S1a). Thus the bulk of the WCMC37 cell culture was silenced even in the absence of differentiation. It is possible that the differences in this study and that of Colak et al. [18] reflects differences in the passage number of the cells used. However, the large *EcoRI*-*Eag1* fragment seen in southern blots in the previous reports (Fig. S1 of [18], Fig. 3b of [46], and Fig. 2a and Fig. S1 of [33]) would be consistent with even early passage cultures of WCMC37 containing significant numbers of cells in which the FM allele was already silenced.

Propagation of WCMC37 cells for 22 passages showed no clear evidence of instability (Additional file 2: Figure S2a). However, of 6 individual sub-populations that we isolated from this culture, all had undergone contractions (Fig. 2a). It may be that as with the PM lines, the stresses associated with growth at low cell density favor more rapid growth and thus the loss of repeats that we have shown to impede DNA replication [15, 44].

### A threshold for stable methylation is seen in FX ESCs

**Fig. 3:**
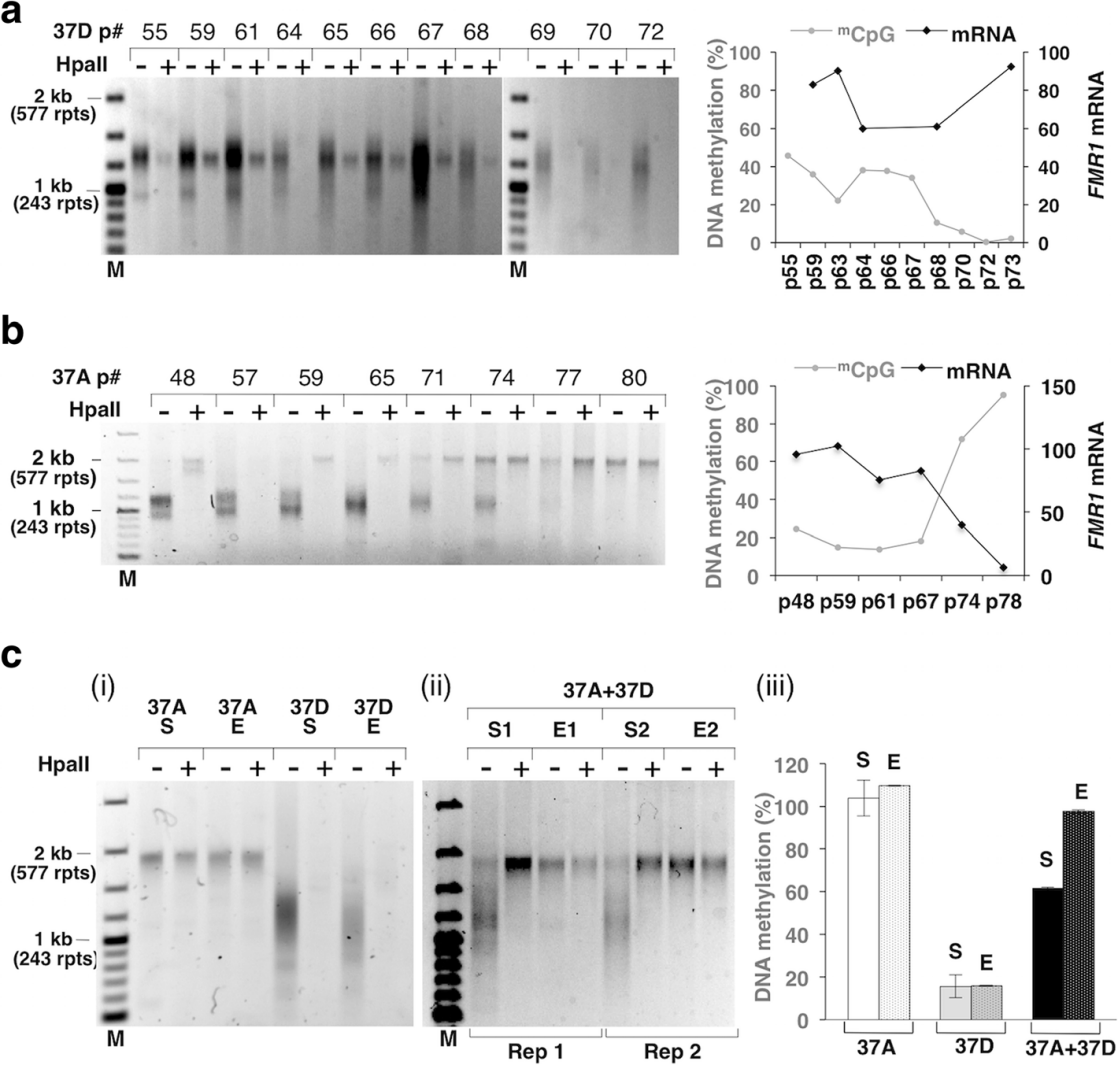
Selective growth advantage of cells carrying methylated *FMR1* alleles with large CGG repeats. **a**–**c** The repeat size, methylation status, and *FMR1* mRNA levels of the indicated cultures were monitored as described in the “Methods” section. The “+” and “−” signs indicate the presence or absence of predigestion by the methylation-sensitive restriction enzyme, HpaII. M, 100-bp DNA size ladder and rpts, CGG repeats. **a**, **b** Data for 37D and 37A lineages that were maintained in culture for extended periods of time. The DNA methylation status is indicated by the *grey line* and *symbols* in the right hand panel, and the mRNA level is indicated by the *black line* and *symbols*. **c** Growth of methylated 37A and unmethylated 37D cells. Late passage 37A cells that were completely methylated and late passage 37D cells that were unmethylated were either grown separately (*i*) or in a ~1:1 mixture (*ii*) for ~20 passages. *S* refers to the cells at the start of the experiment and *E* to the cells at the end of the experiment. Data for the mixed cultures are shown from two independent experiments (Rep 1 and Rep 2). Panel (*iii*) shows the DNA methylation for each set of cultures at the start and end of the experiment as an average from two experiments and the *error bars* indicate standard deviation

Interestingly, contracted alleles with >400 repeats like those in lines 37E, 37F and the largest allele in 37C remained hypermethylated, while alleles with <400 repeats like those in 37A, 37B, 37C, and 37D became unmethylated or partially unmethylated (Fig. 2a, c). Further propagation of 37D, which contains a mixture of similarly sized methylated and unmethylated alleles all <400 repeats (Fig. 2a), resulted in the complete loss of all traces of the methylated allele (Fig. 3a). This methylation loss may reflect a threshold for methylation maintenance in these cells of ~400 repeats. While it is possible that this methylation threshold varies between different cell lines or FM carriers, it should be noted that the first FX ESC line isolated, HEFX had a repeat number of ~300 and was unmethylated [16]. Furthermore, many other FX ESC lines have since been described that contain a mixture of methylated and unmethylated alleles with the methylated alleles having ~400 or more repeats and the unmethylated ones having fewer than ~400 repeats [17, 33]. Additionally, many carriers of UFM alleles also have <400 repeats [5, 8, 10, 47].

Thus, our data, in combination with data from multiple UFM carriers, raise the possibility that the threshold for stable methylation is higher than generally assumed. If so, then carriers of UFM alleles may not have second site mutations that prevent silencing but rather do not methylate their FM allele because their repeat number is below the methylation threshold.

The loss of methylation in FX ESCs with <400 repeats may reflect the more dynamic methylation in ESCs relative to differentiated cells [48, 49]. This dynamic nature may result from exclusion of the methyltransferase responsible for maintenance methylation, DNMT1, from the nucleus during early embryonic development [50], in combination with elevated expression of ten-eleven translocation (Tet) family of proteins responsible for active DNA demethylation [51]. We speculate that while contracted alleles are rapidly demethylated in ESCs when their repeat number drops below ~400, alleles that contract in differentiated cells may not lose their methylation as easily, because active demethylation is reduced and these cells rely more heavily on the clonal propagation of epigenetic memory via maintenance methylation [49]. This may account for those FM carriers with methylated alleles of 200–400 repeats. It could also account for a case we recently described of a male with a broad range of heterogeneous unmethylated FM alleles and two methylated PM alleles [40], as well as those individuals who inherit FM alleles that subsequently lose all of their repeats by contraction yet retain DNA methylation [52].

While unmethylated alleles were unstable showing both small increases and decreases in repeat number, no evidence was seen of the large step-wise increase in repeat number observed for stem cells from patients with FRDA and DM1 [20–24]. This contrasts with what is seen in FRDA stem cells where alleles with 323 repeats gained 9.6 GAA/TTC repeats with each passage and alleles with 433 repeats gained 13.3 repeats [43]. The instability that we observed is more reminiscent of microsatellite instability (MSI) where repeats are as likely to be lost as gained. Work in a mouse model suggests that MSI is likely to arise via a very different mechanism from expansion since mismatch repair proteins protect against MSI, while they are required for expansion [53–55]. Retrospective examination of data from a previously published paper shows a very similar MSI-like pattern of instability for WCMC37 ESCs and a second FX ESC line, SI-214 [33]. In this report, the cells were grown in conditions similar to those used in the studies of FRDA and DM1 iPSCs where expansions were seen [33]. Thus, the failure to see expansion may not be related to the particular growth conditions used. Furthermore, as seen in Additional file 2: Figure S2b, we saw no evidence of expansion when we treated the WCMC37 cells with caffeine, an inhibitor of the ATR DNA damage response pathway, or KU55933, an inhibitor of the ATM DNA damage response pathway. These two pathways have been implicated in the expansion process in a mouse model [56, 57]. Thus, our data suggest either that expansion in FXS ESCs occurs at a much lower frequency than it does in diseases like FRDA or that it occurs primarily in the oocyte and/or in the very early embryo.

In the case of the 37D lineage, the methylated and unmethylated alleles were similar in size. This would be consistent with the unmethylated alleles having arisen from the methylated allele after contraction had occurred. Thus, it may be that unlike expansions that require transcriptionally competent chromatin [2, 30, 58], contractions can occur from methylated alleles.

### Large active alleles may be at a selective disadvantage

One WCMC37-derived line, 37A, did initially contain a mixture of larger methylated FM alleles and smaller unmethylated ones (Figs. 2a and 3b). However, the largest methylated FM allele was the same size as the largest allele seen in the parental culture. Thus, it is likely that this allele represents an allele present in the original population rather than an expansion product. The culture initially showed ~25 % methylation (Fig. 3b). However, on extended propagation the smaller unmethylated allele disappeared completely, methylation rose to 100 % and no *FMR1* mRNA was produced (Fig. 3b).

To test whether selection could explain how the larger, methylated allele came to dominate the culture, we mixed equal numbers of cells from a late passage 37A culture containing completely methylated alleles, with cells from a late passage of a different WCMC-derived line, 37D that was completely unmethylated. However, after 49 days in co-culture, there was no trace of the smaller, unmethylated alleles in the MS_RPT-PCR assay (Fig. 3c (ii)) or evidence of unmethylated alleles in the qMS-PCR (Fig. 3c (iii)). This is not because the FX allele in 37D cells had also expanded since that same line grown in isolation did not produce such an allele (Fig. 3a, c (i)). Thus, our results suggest that the larger, inactive allele has a growth advantage over the smaller, unmethylated allele as seen with 37A (Fig. 3b).

Transcripts produced from PM alleles are thought to be responsible for FXTAS pathology, either because they sequester essential proteins [59] or because the transcripts support repeat-associated non-AUG (RAN) translation that generates a toxic protein [60]. Symptoms of FXTAS are seen in carriers of UFM alleles [9–11] demonstrating that active FM alleles are similarly deleterious. It is possible that the deleterious effects of active FM alleles are manifest even in ESCs and thus explain the selective advantage of cells with silenced alleles. However, no obvious differences were seen in the viability or growth rate of early passages of 37A containing a mixture of active and silenced alleles and late passage cells containing only silenced alleles in the Alarmablue assay. Nor was there any significant difference in the viability of late passage 37A cells and 37D. However, given the relatively slow rate at which selection occurred, this may not be surprising and more work is need to determine whether this selection reflects the deleterious consequences of expression of unmethylated alleles or genetic changes in the 37A lineage that confers some sort of methylation-independent growth advantage.

### Active FM alleles with <400 repeats do not become silenced on neuronal differentiation

**Fig. 4:**
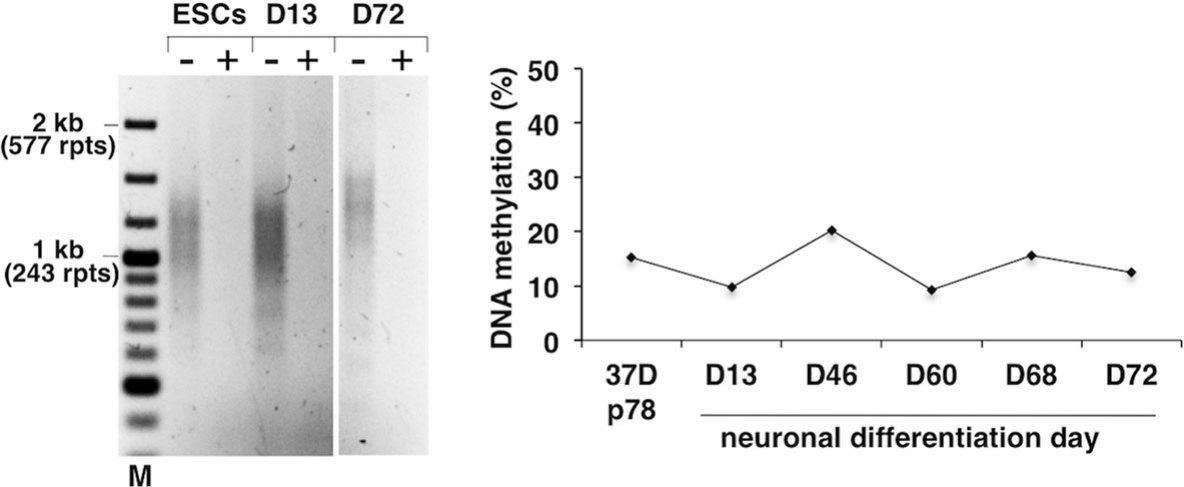
Unmethylated FM alleles do not become silenced on differentiation into neurons. The repeat size, methylation status, and *FMR1* mRNA levels of the indicated cultures were monitored as described in the “Methods” section. The “+” and “−” signs indicate the presence or absence of predigestion by the methylation-sensitive restriction enzyme, HpaII. M, 100-bp DNA size ladder and rpts, CGG repeats. A late passage culture of 37D ESCs containing little, if any methylated alleles, was differentiated into neurons as described in the “Methods” section. Methylation levels were measured by qMS-PCR on the indicated number of days after the initiation of neuronal differentiation. See Additional file 3: Figure S3 for representative images during neuronal differentiation of 37D cells

While differentiation into neurons was associated with high levels of cell death in two independent trials, no evidence of *FMR1* gene silencing was seen when successful neuronal differentiation was observed (Additional file 3: Figure S3), as evidenced by the fact that the DNA was completely sensitive to HpaII predigestion in the MS_RPT-PCR assay and the qMS-PCR assay showed no change in the percentage of DNA methylation (Fig. 4). Thus, in a cell line derived from progenitor cells with the demonstrated ability to complete the silencing process, active FM alleles with <400 repeats do not become silenced on neuronal differentiation. In previous experiments in which the HEFX cell line, which has ~300 repeats, was differentiated into teratomas, a dramatic decrease in transcription was seen. However, this decrease was associated with just 5 % methylation [16]. Subsequent studies of the differentiation of other FX ESCs into neurons also showed a significant decrease in transcription [61] and/or an increase in heterochromatic marks characteristic of silenced genes [18]. However, these studies used ESC lines with alleles that were at least partially silenced and from the data provided it is not possible to distinguish between de novo gene silencing and selection for silenced alleles that were already in the original ESC population.

## Conclusions

In summary, we have shown that contractions occur in PM iPSCs and in FM ESCs but not the large, progressive, and step-wise increases in repeat number seen in ESCs and iPSCs associated with other repeat expansion diseases [20, 43]. Thus even though stem cells express high levels of mismatch repair proteins that have been suggested to account for expansions seen in stem cells from other repeat expansion disorders [43], this is not sufficient to trigger significant levels of expansion in FX stem cells. This would be consistent with genetic data from humans [19] and mouse models [27] that suggest that the expansions that give rise to FXS occur in the oocyte. Since oocytes do not divide, this would support a model in which expansions result from aberrant DNA repair or recombination rather than a problem with DNA replication. This idea would be compatible with work in mice that suggests that expansion occurs as a result of the interaction of mismatch repair proteins with base excision repair proteins [27, 53]. Some of the contractions we observe likely arise from alleles that are methylated. Since expansions do not occur from methylated alleles [29, 30], this supports the hypothesis that expansions and contractions occur by different mechanisms [32].

We have also shown that there is a threshold for methylation stability in the WCMC37 ESC line of ~400 repeats and that FM alleles with <400 repeats that are unmethylated in ESCs do not become re-methylated either on propagation as ESCs or on differentiation into neurons. Since many UFM alleles have <400 repeats [5, 8, 10, 47], such a methylation threshold may explain why they escape methylation. R-loops formed by the repeat tract have recently been suggested to play a role in *FMR1* gene silencing [18, 62]. It is possible that 400 repeats represents the minimum number of repeats required to produce an R-loop that is large enough or stable enough to trigger silencing. Mice with FM alleles with <400 repeats also do not show silencing [63, 64]. If mice have a similar methylation threshold as humans, a mouse model with >400 repeats may be useful for recapitulating early developmental events in FXS.

Dynamic methylation in ESCs and the clonal propagation of methylation in differentiated cells might result in contractions having different consequences depending on whether they occur early or late in embryonic development. Contractions to <400 repeats in the very early embryo may be more likely to result in unmethylated alleles that could contribute to the risk of FXTAS symptoms. In contrast, contractions occurring later may result in alleles that retain their DNA methylation and thus contribute to the symptoms of FXS.

Our data also raises the possibility that selection favors the accumulation of large, silenced alleles. If true, this would make it difficult to distinguish between differentiation-induced silencing [18] and selection for silenced alleles in an initially heterogeneous cell population. It would also have potential implications for both PM and UFM carriers. Both PM and UFM carriers are at risk of FXTAS [9–11], and our data suggest that the deleterious effects of such alleles may begin to be apparent very early in life.

## Additional files

Additional file 1: Figure S1. Analysis of premutation iPSCs and full mutation ESCs. *a* Immunostaining for pluripotency markers in SC120 and HT14 iPSCs and WCMC37 ESCs. Cells were grown in 24-well plates, fixed and stained for the indicated pluripotency markers (red) as described in the supplemental experimental procedure. Nuclei were stained with DAPI (blue). Scale bar: 200 μm. *b* SC120 iPSCs were differentiated into neurons and stained for neuronal markers. SC120 neural progenitor cells (NPC) were stained at passage 2 with Nestin (green) and Sox1 (red). Scale bar: 200 μm. SC120 neurons were stained at 4 weeks of differentiation with Map2 (red) and TuJ1 (red). Nuclei were stained with DAPI (blue). Scale bar: 100 μm. *c* DNA methylation at the FMR1 promoter was analyzed by qMS-PCR in SC120 iPSCs, NPCs, and neurons at indicated weeks (wk) of differentiation. *d* Immunostaining for FMRP (red) was done in indicated cell lines. The percentage of FMRP-positive cells is indicated under each panel. In SC120 iPSCs, all the cells were positive for FMRP expression but the intensity was much reduced compared to the control H1 cells. Scale bar: 100 μm. *e* Western blot for FMRP levels in indicated cell lines. β-Actin is used as loading control. *f* Western blot analysis for MSH2, MSH3, and MSH6 proteins in stem cells and fibroblasts was done as described in the supplemental experimental procedures. β-Actin is used as loading control. (TIFF 11721 kb)
Additional file 2: Figure S2. CGG-repeat size in FX ESCs. *a* WCMC37 cells were grown in culture for 22 passages and CGG-repeat size was analyzed by RPT-PCR. *b* 37D p70 cells were treated with either 1 mM caffeine or 10 μM of the ATM kinase inhibitor KU55933 for 24 h and then grown in drug-free medium for the next 3 days. Cells were passaged on the third day and treated with the drug again for 24 h for a total of three treatments. After the last treatment, cells were grown for 3 days in drug-free medium and harvested for DNA. The CGG-repeat size was analyzed by RPT-PCR. (TIFF 11721 kb)
Additional file 3: Figure S3. Neuronal differentiation of 37D ESCs. Top panel shows the immunostaining for pluripotency markers (red) in 37D cells that were used for the neuronal differentiation experiments shown in Fig. 2d. The differentiating cells (bottom panels) were stained for neuronal markers at indicated days. Nestin (red), Map2 (red), TuJ1 (green). Nuclei were stained with DAPI (blue). Scale bar: 200 μm. (TIFF 11721 kb)

## Abbreviations

ChIP: Chromatin immunoprecipitation
ESCs: Embryonic stem cells
FM: Full mutation
FMR1: Fragile X mental retardation 1
FXS: Fragile X syndrome
FXTAS: Fragile X-associated tremor/ataxia syndrome
iPSCs: Induced pluripotent stem cells
MS_RPT-PCR: Methylation-specific repeat PCR
PM: Premutation
qMS-PCR: Quantitative methylation-specific PCR
qRT-PCR: Quantitative reverse transcription PCR
RPT-PCR: Repeat PCR
UFM: Unmethylated full mutation
