# Supplementary figures and images for: CGG-repeat dynamics and FMR1 gene silencing in fragile X syndrome stem cells and stem cell-derived neurons
Source: Mol Autism. 2016 Oct 6;7:42. doi: 10.1186/s13229-016-0105-9 (PMC5053128; doi:10.1186/s13229-016-0105-9)

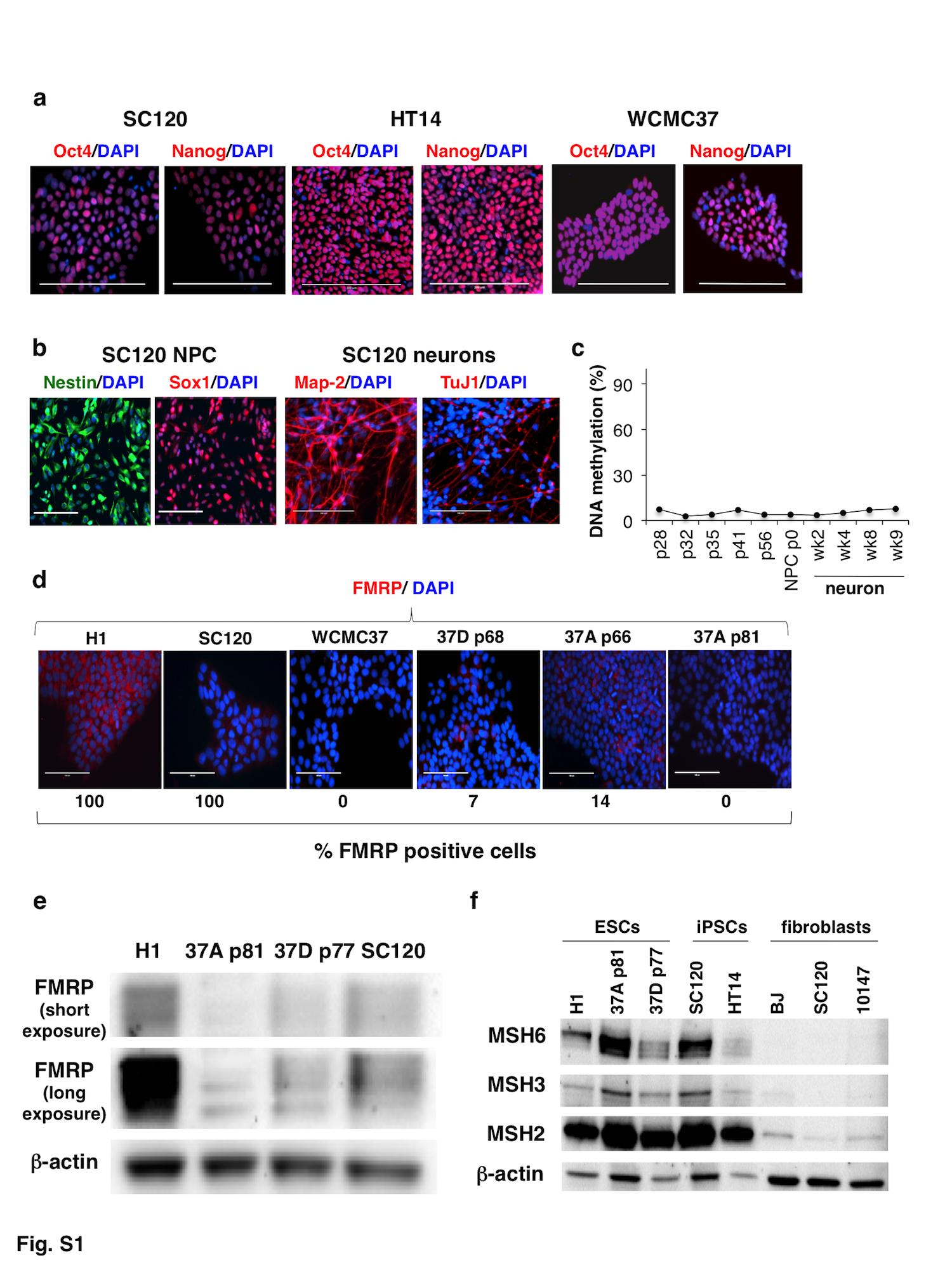

Supplement: Additional file 1: Figure S1. — Analysis of premutation iPSCs and full mutation ESCs. a Immunostaining for pluripotency markers in SC120 and HT14 iPSCs and WCMC37 ESCs. Cells were grown in 24-well plates, fixed and stained for the indicated pluripotency markers (red) as described in the supplemental experimental procedure. Nuclei were stained with DAPI (blue). Scale bar: 200 μm. b SC120 iPSCs were differentiated into neurons and stained for neuronal markers. SC120 neural progenitor cells (NPC) were stained at passage 2 with Nestin (green) and Sox1 (red). Scale bar: 200 μm. SC120 neurons were stained at 4 weeks of differentiation with Map2 (red) and TuJ1 (red). Nuclei were stained with DAPI (blue). Scale bar: 100 μm. c DNA methylation at the FMR1 promoter was analyzed by qMS-PCR in SC120 iPSCs, NPCs, and neurons at indicated weeks (wk) of differentiation. d Immunostaining for FMRP (red) was done in indicated cell lines. The percentage of FMRP-positive cells is indicated under each panel. In SC120 iPSCs, all the cells were positive for FMRP expression but the intensity was much reduced compared to the control H1 cells. Scale bar: 100 μm. e Western blot for FMRP levels in indicated cell lines. β-Actin is used as loading control. f Western blot analysis for MSH2, MSH3, and MSH6 proteins in stem cells and fibroblasts was done as described in the supplemental experimental procedures. β-Actin is used as loading control. (TIFF 11721 kb) [file 13229_2016_105_MOESM1_ESM.tiff]

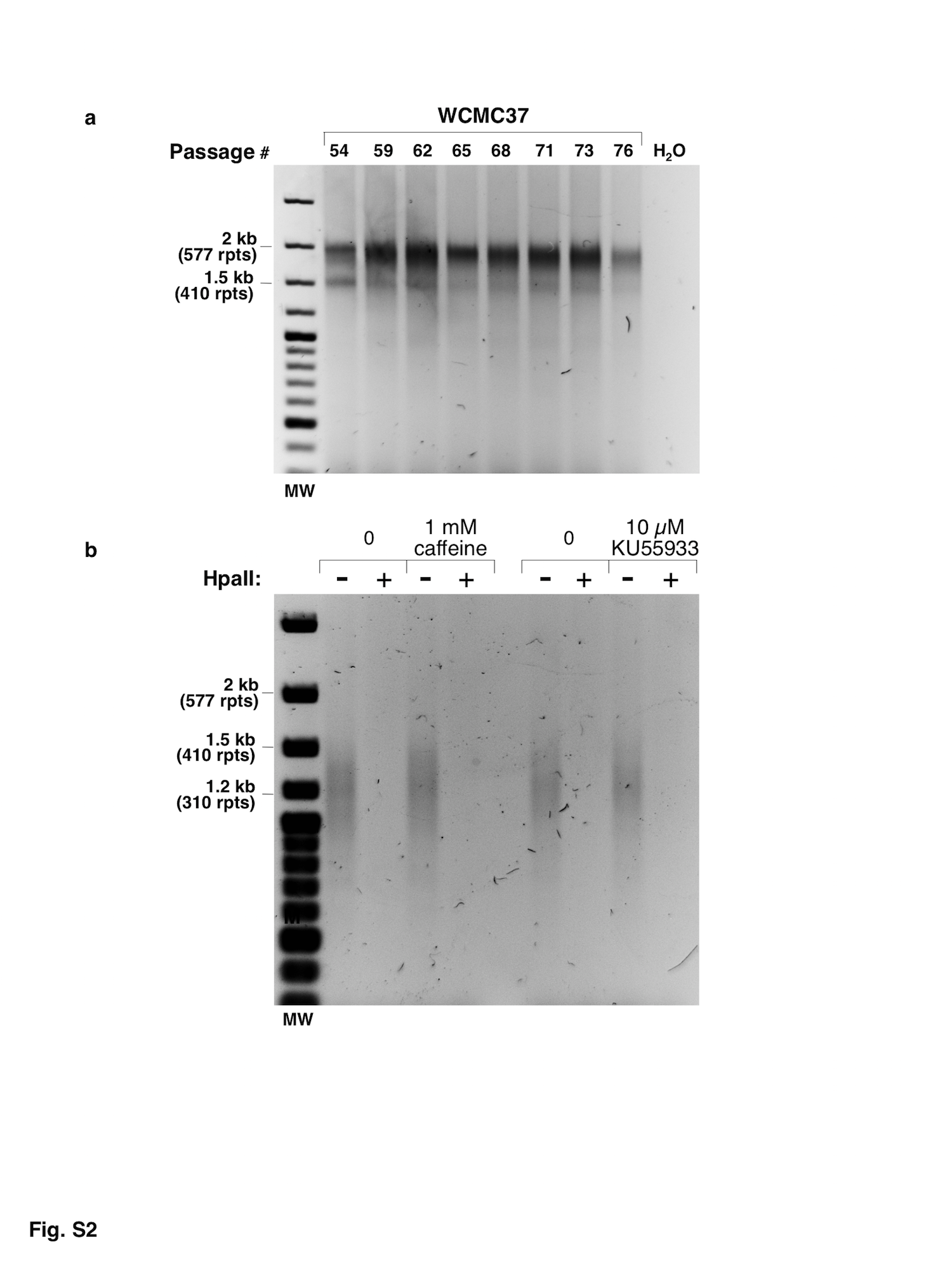

Supplement: Additional file 2: Figure S2. — CGG-repeat size in FX ESCs. a WCMC37 cells were grown in culture for 22 passages and CGG-repeat size was analyzed by RPT-PCR. b 37D p70 cells were treated with either 1 mM caffeine or 10 μM of the ATM kinase inhibitor KU55933 for 24 h and then grown in drug-free medium for the next 3 days. Cells were passaged on the third day and treated with the drug again for 24 h for a total of three treatments. After the last treatment, cells were grown for 3 days in drug-free medium and harvested for DNA. The CGG-repeat size was analyzed by RPT-PCR. (TIFF 11721 kb) [file 13229_2016_105_MOESM2_ESM.tiff]

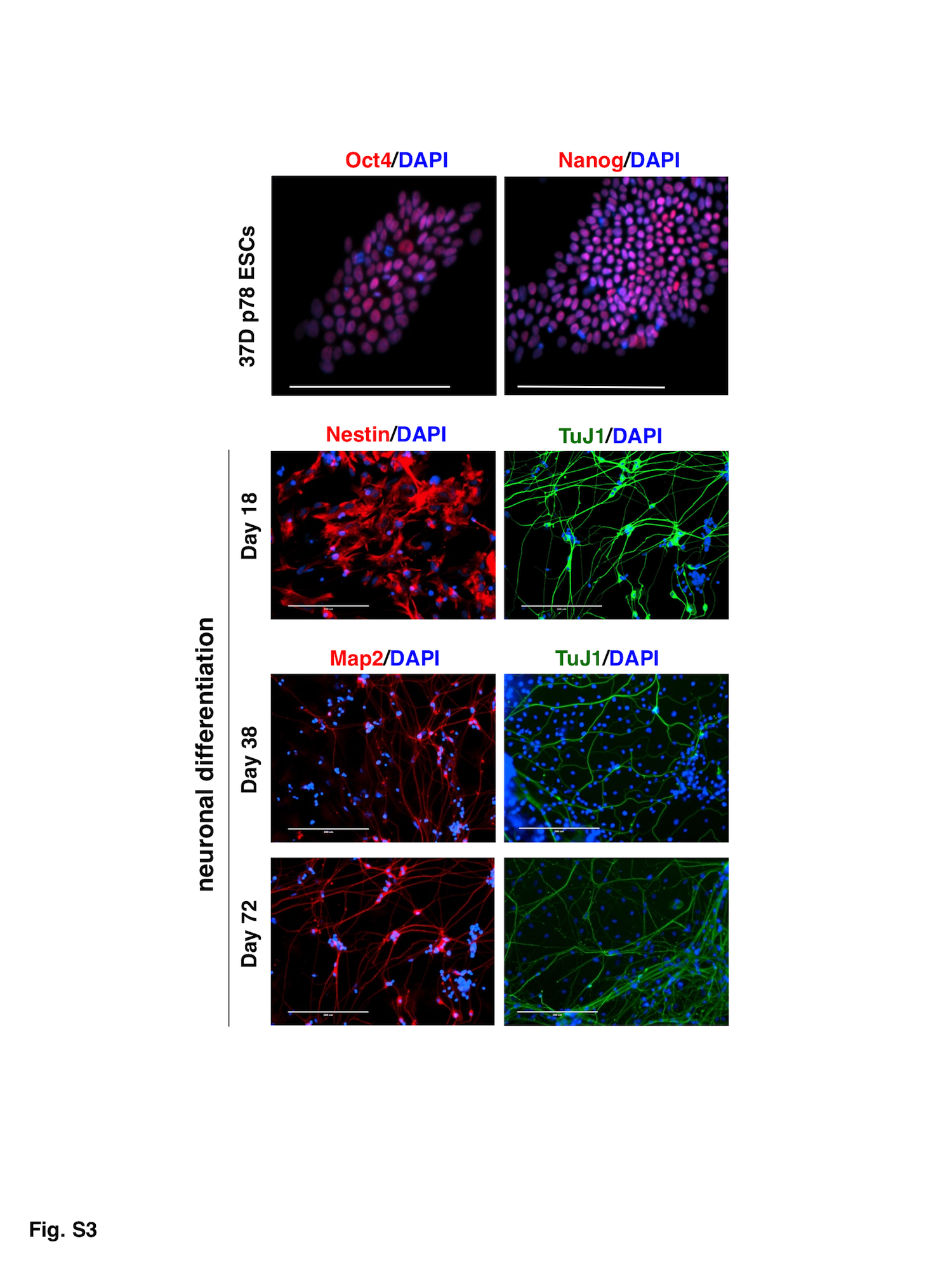

Supplement: Additional file 3: Figure S3. — Neuronal differentiation of 37D ESCs. Top panel shows the immunostaining for pluripotency markers (red) in 37D cells that were used for the neuronal differentiation experiments shown in Fig. 2d. The differentiating cells (bottom panels) were stained for neuronal markers at indicated days. Nestin (red), Map2 (red), TuJ1 (green). Nuclei were stained with DAPI (blue). Scale bar: 200 μm. (TIFF 11721 kb) [file 13229_2016_105_MOESM3_ESM.tiff]
